# Supplementary material for: Residual effect of defeated stripe rust resistance genes/QTLs in bread wheat against prevalent pathotypes of Puccinia striiformis f. sp. tritici
Source: PLoS One. 2022 Apr 1;17(4):e0266482. doi: 10.1371/journal.pone.0266482 (PMC8975100; doi:10.1371/journal.pone.0266482)
Supplement: S1 Table — (DOCX) [file pone.0266482.s001.docx]

**S1 Table. Avirulence/ virulence formula of predominant Indian wheat rust pathotypes used in the present study**

| **Pathotype** | **Virulence** | **Avirulence** |
| --- | --- | --- |
| 238S119 | *Yr2, Yr3, Yr6, Yr7, Yr8, Yr9, Yr11, Yr12, Yr17, Yr18, Yr19, Yr21, Yr22, Yr23, Yr24, Yr25, Yr26, Yr27, Yrri, Yrso, Yrsd* | *Yr1, Yr4, Yr5, Yr10, Yr13, Yr14, Yr15, Yr16, Yrsk, YrA* |
| 110S119 | *Yr2, Yr3, Yr4, Yr6, Yr7, Yr8, Yr9, Yr11, Yr12, Yr17, Yr18, Yr19, Yr21, Yr22, Yr23, Yr25, YrA, Yrso* | *Yr1, Yr5, Yr10, Yr13, Yr14, Yr15, Yr16, Yr24, Yr26, Yrsp, Yrsk* |
| 46S119 | *Yr2, Yr3, Yr4, Yr6, Yr7, Yr8, Yr9, Yr17, Yr18, Yr19, Yr21, Yr22, Yr23, Yr25, YrA, Yrsd, Yrso* | *Yr1, Yr5, Yr10, Yr11, Yr12, Yr13, Yr14, Yr15, Yr16, Yr24, Yr26, Yrsp, Yrso, Yrsk* |
| 78S84 | *Yr2, Yr3a, Yr4A, Yr6, Yr7, Yr8, Yr9, Yr12, Yr19, Yr27,YrSk, YrSu, Yr31* | *Yr1, Yr 3b, Yr4b, Yr5, Yr10, Yr11, Yr14, Yr15, Yr17,, Yr18, Yr24/26, Yr28, Yr29, YrSD, Riebesel 147/51(Yr2,9,+)* |
