# Supplementary material for: Residual effect of defeated stripe rust resistance genes/QTLs in bread wheat against prevalent pathotypes of Puccinia striiformis f. sp. tritici
Source: PLoS One. 2022 Apr 1;17(4):e0266482. doi: 10.1371/journal.pone.0266482 (PMC8975100; doi:10.1371/journal.pone.0266482)
Supplement: S2 Table — (DOC) [file pone.0266482.s002.doc]

**S2 Table. Primer name, sequence of three stripe rust resistance genes (*Yr9*, *Yr17* and *Yr27*)**

| **Gene** | **Marker name** | **Primer sequence** | **Reference** |
| --- | --- | --- | --- |
| *Yr9* | Xgwm582-1B | AAGCACTACGAAAATATGAC  TCTTAAGGGGTGTTATCATA | [21] |
| *Yr17* | VENTRIUP  LN2  Yr17neg | AGGGGCTACTGACCAAGGCT TGCAGCTACAGCAGTATGTACACAAAA GATCCATGACGCGCATTT | [22] |
| *Yr27* | Xcdo405-2B/Xbcd152-2B | GTAAAACGACGGCCACT  AACAGCTATGACCATG | [10] |
