# Supplementary material for: Residual effect of defeated stripe rust resistance genes/QTLs in bread wheat against prevalent pathotypes of Puccinia striiformis f. sp. tritici
Source: PLoS One. 2022 Apr 1;17(4):e0266482. doi: 10.1371/journal.pone.0266482 (PMC8975100; doi:10.1371/journal.pone.0266482)
Supplement: S3 Table — (DOC) [file pone.0266482.s003.doc]

**S3 Table. Disease reaction in field nursery and epidemiological parameters on extreme phenotypic categories of cross PBW 621× PBW 343**

| **Crop Season** | **2016-17** | | | | | | | **2017-18** | | | | | | |
| --- | --- | --- | --- | --- | --- | --- | --- | --- | --- | --- | --- | --- | --- | --- |
| **Extreme**  **category No.** | **Disease reaction** | | **Mean values of different epidemiological parameters** for F1 and F leaf** | | | | | **Disease reaction** | | **Mean values of different epidemiological parameters for F1 and F leaf** | | | | |
|  | **P*** | **F*** | **AUDPC** | **rAUDPC** | **FRS** | **CI** | **Infection rate (r)** | **P** | **F** | **AUDPC** | **rAUDPC** | **FRS** | **CI** | **Infection rate (r)** |
| 1 | 60S | 60S | 675 | 61.3 | 60 | 60 | 0.25 | 60S | 60S | 800 | 75.2 | 60 | 60 | 0.21 |
| 2 | 60S | 40S | 625 | 55.8 | 50 | 50 | 0.21 | 60S | 40S | 625 | 55.8 | 50 | 50 | 0.21 |
| 3 | 60S | 40S | 625 | 55.8 | 50 | 50 | 0.21 | 60S | 60S | 800 | 75.2 | 60 | 60 | 0.21 |
| 4 | 60S | 10S | 450 | 36.3 | 35 | 35 | 0.24 | 60S | 40S | 625 | 55.8 | 50 | 50 | 0.21 |
| 5 | 20S | 10S | 162.5 | 14.2 | 15 | 15 | 0.23 | 10S | 5S | 62.5 | 5.2 | 7.5 | 7.5 | 0.23 |
| 6 | 20S | 5S | 125 | 10.0 | 12.5 | 12.5 | 0.18 | 20S | 5MS | 125 | 10.0 | 12.5 | 12 | 0.17 |
| 7 | 5S | 0 | 12.5 | 0.96 | 2.5 | 2.5 | 0.09 | 10S | 5S | 62.5 | 5.2 | 7.5 | 7.5 | 0.23 |
| 8 | 20S | 5S | 125 | 10.0 | 12.5 | 12.5 | 0.18 | 20MS | 5S | 125 | 10.0 | 12.5 | 10.5 | 0.18 |
| 9 | 20MS | 5MS | 125 | 10.0 | 12.5 | 10 | 0.16 | 40S | 5S | 237.5 | c18.7 | 22.5 | 22.5 | 0.20 |
| 10 | 60S | 40S | 625 | 55.8 | 50 | 50 | 0.21 | 60S | 40S | 625 | 55.8 | 50 | 50 | 0.21 |
| 11 | 10MS | 5MS | 125 | 10.9 | 7.5 | 6 | 0.11 | 10S | 5MS | 62.5 | 5.2 | 7.5 | 7 | 0.22 |
| 12 | 60S | 20S | 512.5 | 43.3 | 40 | 40 | 0.19 | 60S | 10S | 450 | 36.3 | 35 | 35 | 0.24 |
| 13 | 60S | 60S | 800 | 75.2 | 60 | 60 | 0.21 | 60S | 60S | 800 | 75.2 | 60 | 60 | 0.21 |
| 14 | 20MS | 0 | 112.5 | 8.7 | 10 | 8 | 0.09 | 20S | 0 | 112.5 | 8.7 | 10 | 10 | 0.09 |
| 15 | 20MS | 5MS | 125 | 10.0 | 12.5 | 10 | 0.17 | 10MS | 0 | 50 | 3.8 | 5 | 4 | 0.12 |
| 16 | 60S | 20S | 512.5 | 43.3 | 40 | 40 | 0.19 | 60S | 40S | 625 | 55.8 | 50 | 50 | 0.21 |
| 17 | 20S | 0 | 112.5 | 8.7 | 10 | 10 | 0.09 | 20S | 5S | 125 | 10.0 | 12.5 | 12.5 | 0.18 |
| 18 | 60S | 20S | 625 | 55.8 | 50 | 50 | 0.21 | 60S | 40S | 625 | 55.8 | 50 | 50 | 0.21 |
| 19 | 60S | 20S | 512.5 | 43.3 | 40 | 40 | 0.19 | 60S | 40S | 625 | 55.8 | 50 | 50 | 0.21 |
| 20 | 20MS | 0 | 112.5 | 8.7 | 10 | 8 | 0.09 | 10S | 0 | 50 | 3.8 | 5 | 5 | 0.14 |

***P (Penultimate leaf)-one leaf below the flag leaf, F-flag leaf (In all the parameters mean value of both the leaves is given)**

****AUDPC-area under disease progress curve, rAUDPC-relative area under disease progress curve, FRS-final rust severity, CI-cofficient of infection**
