# Supplementary material for: Residual effect of defeated stripe rust resistance genes/QTLs in bread wheat against prevalent pathotypes of Puccinia striiformis f. sp. tritici
Source: PLoS One. 2022 Apr 1;17(4):e0266482. doi: 10.1371/journal.pone.0266482 (PMC8975100; doi:10.1371/journal.pone.0266482)
Supplement: S4 Table — (DOC) [file pone.0266482.s004.doc]

**S4 Table. Disease reaction in field nursery and epidemiological parameters on extreme phenotypic category of cross PBW 621× HD 2967**

| **Crop season** | **2016-17** | | | | | | | **2017-18** | | | | | | |
| --- | --- | --- | --- | --- | --- | --- | --- | --- | --- | --- | --- | --- | --- | --- |
| **Extreme**  **category No.** | **Disease reaction** | | **Mean values of different epidemiological parameters** for F1 and F leaf** | | | | | **Disease reaction** | | **Mean values of different epidemiological parameters for F1 and F leaf** | | | | |
|  | **P*** | **F*** | **AUDPC** | **rAUDPC** | **FRS** | **CI** | **Infection rate (r)** | **P** | **F** | **AUDPC** | **rAUDPC** | **FRS** | **CI** | **Infection rate (r)** |
| 1 | 5S | 0 | 12.5 | 1.0 | 2.5 | 2.5 | 0.1 | 10S | 0 | 50.0 | 3.8 | 5.0 | 5.0 | 0.1 |
| 2 | 5S | 0 | 12.5 | 1.0 | 2.5 | 2.5 | 0.1 | 5S | 0 | 12.5 | 1.0 | 2.5 | 2.5 | 0.1 |
| 3 | 60S | 40S | 625.0 | 55.8 | 50.0 | 50.0 | 0.2 | 60S | 40S | 625.0 | 55.8 | 50.0 | 50.0 | 0.2 |
| 4 | 40S | 0 | 225.0 | 17.3 | 20.0 | 20.0 | 0.1 | 60S | 10S | 450.0 | 36.3 | 35.0 | 35.0 | 0.2 |
| 5 | 60S | 10S | 450.0 | 36.3 | 35.0 | 35.0 | 0.2 | 60S | 5S | 412.5 | 32.2 | 32.5 | 32.5 | 0.2 |
| 6 | 60S | 20S | 512.5 | 43.3 | 40.0 | 40.0 | 0.2 | 60S | 20S | 512.5 | 43.3 | 40.0 | 40.0 | 0.2 |
| 7 | 40S | 20S | 325.0 | 28.4 | 30.0 | 30.0 | 0.3 | 40S | 20S | 337.5 | 29.8 | 30.0 | 30.0 | 0.2 |
| 8 | 5S | 0 | 12.5 | 1.0 | 2.5 | 2.5 | 0.1 | 5S | 0 | 12.5 | 1.0 | 2.5 | 2.5 | 0.1 |
| 9 | 60S | 20S | 512.5 | 43.3 | 40.0 | 40.0 | 0.2 | 60S | 40S | 625.0 | 55.8 | 50.0 | 50.0 | 0.2 |
| 10 | 40S | 20MS | 337.5 | 29.8 | 30.0 | 28.0 | 0.3 | 40S | 20MS | 325.0 | 28.4 | 30.0 | 28.0 | 0.3 |
| 11 | 60S | 40S | 625.0 | 55.8 | 50.0 | 50.0 | 0.1 | 60S | 40S | 625.0 | 55.8 | 50.0 | 50.0 | 0.2 |
| 12 | 10MS | 0 | 50.0 | 3.8 | 5.0 | 4.0 | 0.1 | 10S | 0 | 50.0 | 3.8 | 5.0 | 5.0 | 0.1 |
| 13 | 60S | 60S | 675.0 | 61.3 | 60.0 | 60.0 | 0.3 | 60S | 40S | 625.0 | 55.8 | 50.0 | 50.0 | 0.2 |
| 14 | 60S | 5S | 412.5 | 32.2 | 32.5 | 32.5 | 0.2 | 60S | 40S | 625.0 | 55.8 | 50.0 | 50.0 | 0.2 |
| 15 | 20S | 5S | 125.0 | 10.0 | 12.5 | 12.5 | 0.2 | 20S | 5S | 125.0 | 10.0 | 12.5 | 12.5 | 0.2 |
| 16 | 20MS | 0 | 112.5 | 8.7 | 10.0 | 8.0 | 0.1 | 20MS | 0 | 112.5 | 8.7 | 10.0 | 8.0 | 0.1 |
| 17 | 5S | 0 | 12.5 | 1.0 | 2.5 | 2.5 | 0.1 | 5S | 0 | 12.5 | 1.0 | 2.5 | 2.5 | 0.1 |
| 18 | 5MS | 0 | 12.5 | 1.0 | 2.5 | 2.0 | 0.1 | 10S | 5S | 62.5 | 5.2 | 7.5 | 7.5 | 0.2 |
| 19 | 5S | 0 | 12.5 | 1.0 | 2.5 | 2.5 | 0.1 | 5S | 0 | 12.5 | 1.0 | 2.5 | 2.5 | 0.1 |
| 20 | 60S | 10S | 450.0 | 36.3 | 35.0 | 35.0 | 0.2 | 60S | 60S | 800.0 | 75.2 | 60.0 | 60.0 | 0.2 |
| 21 | 5MS | 0 | 37.5 | 2.9 | 2.5 | 2.0 | 0.1 | 10MS | 0 | 50.0 | 3.8 | 5.0 | 4.0 | 0.1 |
| 22 | 40S | 10S | 275.0 | 22.9 | 25.0 | 25.0 | 0.2 | 60S | 40S | 625.0 | 55.8 | 50.0 | 50.0 | 0.2 |
| 23 | 40S | 5S | 237.5 | 18.7 | 22.5 | 22.5 | 0.2 | 40S | 0 | 225.0 | 17.3 | 20.0 | 20.0 | 0.1 |
| 24 | 20S | 5S | 125.0 | 10.0 | 12.5 | 12.5 | 0.2 | 20S | 0 | 112.5 | 8.7 | 10.0 | 10.0 | 0.1 |
| 25 | 5MS | 0 | 37.5 | 2.9 | 2.5 | 2.0 | 0.1 | 10S | 0 | 50.0 | 3.8 | 5.0 | 5.0 | 0.1 |
| 26 | 60S | 40S | 625.0 | 55.8 | 50.0 | 50.0 | 0.2 | 60S | 40S | 625.0 | 55.8 | 50.0 | 50.0 | 0.2 |
| 27 | 5MS | 0 | 37.5 | 2.9 | 2.5 | 2.0 | 0.1 | 5S | 0 | 12.5 | 1.0 | 2.5 | 2.5 | 0.1 |
| 28 | 5MS | 0 | 37.5 | 2.9 | 2.5 | 2.0 | 0.1 | 5S | 0 | 12.5 | 1.0 | 2.5 | 2.5 | 0.1 |
| 29 | 10MS | 0 | 50.0 | 3.8 | 5.0 | 4.0 | 0.1 | 20S | 5MS | 125.0 | 10.0 | 12.5 | 12.0 | 0.2 |
| 30 | 5S | 0 | 37.5 | 2.9 | 2.5 | 2.5 | 0.1 | 20S | 0 | 112.5 | 8.7 | 10.0 | 10.0 | 0.1 |
| 31 | 60S | 40S | 625.0 | 55.8 | 50.0 | 50.0 | 0.2 | 60S | 40S | 625.0 | 55.8 | 50.0 | 50.0 | 0.2 |
| 32 | 10MS | 5S | 62.5 | 5.2 | 7.5 | 6.5 | 0.2 | 5S | 0 | 12.5 | 1.0 | 2.5 | 2.5 | 0.1 |
| 33 | 5S | 0 | 37.5 | 2.9 | 2.5 | 2.5 | 0.1 | 20S | 0 | 112.5 | 8.7 | 10.0 | 10.0 | 0.1 |
| 34 | 5S | 0 | 12.5 | 1.0 | 2.5 | 2.5 | 0.1 | 10S | 0 | 50.0 | 3.8 | 5.0 | 5.0 | 0.1 |
| 35 | 5S | 0 | 37.5 | 2.9 | 2.5 | 2.5 | 0.1 | 10S | 0 | 50.0 | 3.8 | 5.0 | 5.0 | 0.1 |
| 36 | 5S | 0 | 12.5 | 1.0 | 2.5 | 2.5 | 0.1 | 20S | 0 | 112.5 | 8.7 | 10.0 | 10.0 | 0.1 |
| 37 | 10S | 0 | 50.0 | 3.8 | 5.0 | 5.0 | 0.1 | 10S | 0 | 50.0 | 3.8 | 5.0 | 5.0 | 0.1 |
| 38 | 10S | 0 | 50.0 | 3.8 | 5.0 | 5.0 | 0.1 | 10S | 5S | 62.5 | 5.2 | 7.5 | 7.5 | 0.2 |
| 39 | 40S | 10S | 275.0 | 22.9 | 25.0 | 25.0 | 0.2 | 40S | 5S | 237.5 | 18.7 | 22.5 | 22.5 | 0.2 |
| 40 | 5S | 0 | 12.5 | 1.0 | 2.5 | 2.5 | 0.1 | 20S | 0 | 112.5 | 8.7 | 10.0 | 10.0 | 0.1 |
| 41 | 10S | 0 | 50.0 | 3.8 | 5.0 | 5.0 | 0.1 | 5S | 0 | 12.5 | 1.0 | 2.5 | 2.5 | 0.1 |
| 42 | 5S | 0 | 12.5 | 1.0 | 2.5 | 2.5 | 0.1 | 5S | 0 | 12.5 | 1.0 | 2.5 | 2.5 | 0.1 |
| 43 | 5S | 0 | 12.5 | 1.0 | 2.5 | 2.5 | 0.1 | 10S | 0 | 50.0 | 3.8 | 5.0 | 5.0 | 0.1 |
| 44 | 5S | 0 | 12.5 | 1.0 | 2.5 | 2.5 | 0.1 | 10S | 0 | 50.0 | 3.8 | 5.0 | 5.0 | 0.1 |
| 45 | 20S | 5S | 125.0 | 10.0 | 12.5 | 12.5 | 0.2 | 5S | 0 | 12.5 | 1.0 | 2.5 | 2.5 | 0.1 |
| 46 | 10S | 5S | 62.5 | 5.2 | 7.5 | 7.5 | 0.2 | 20S | 0 | 112.5 | 8.7 | 10.0 | 10.0 | 0.1 |
| 47 | 20S | 5S | 125.0 | 10.0 | 12.5 | 12.5 | 0.2 | 5S | 0 | 12.5 | 1.0 | 2.5 | 2.5 | 0.1 |
| 48 | 5S | 0 | 12.5 | 1.0 | 2.5 | 2.5 | 0.1 | 20S | 0 | 112.5 | 8.7 | 10.0 | 10.0 | 0.1 |
| 49 | 10S | 0 | 50.0 | 3.8 | 5.0 | 5.0 | 0.1 | 20S | 0 | 112.5 | 8.7 | 10.0 | 10.0 | 0.1 |
| 50 | 60S | 10S | 450.0 | 36.3 | 35.0 | 35.0 | 0.2 | 60S | 40S | 625.0 | 55.8 | 50.0 | 50.0 | 0.2 |
| 51 | 60S | 10S | 450.0 | 36.3 | 35.0 | 35.0 | 0.2 | 60S | 40S | 625.0 | 55.8 | 50.0 | 50.0 | 0.2 |
| 52 | 5S | 0 | 12.5 | 1.0 | 2.5 | 2.5 | 0.1 | 5S | 0 | 12.5 | 1.0 | 2.5 | 2.5 | 0.1 |
| 53 | 5S | 0 | 12.5 | 1.0 | 2.5 | 2.5 | 0.1 | 20S | 5S | 125.0 | 10.0 | 12.5 | 12.5 | 0.2 |
| 54 | 5MS | 0 | 12.5 | 1.0 | 2.5 | 2.0 | 0.1 | 5MS | 0 | 12.5 | 1.0 | 2.5 | 2.0 | 0.1 |
| 55 | 5MS | 0 | 12.5 | 1.0 | 2.5 | 2.0 | 0.1 | 5MS | 0 | 12.5 | 1.0 | 2.5 | 2.0 | 0.1 |
| 56 | 10S | 5S | 62.5 | 5.2 | 7.5 | 7.5 | 0.2 | 20S | 0 | 112.5 | 8.7 | 10.0 | 10.0 | 0.1 |
| 57 | 10S | 5S | 62.5 | 5.2 | 7.5 | 7.5 | 0.2 | 10S | 0 | 50.0 | 3.8 | 5.0 | 5.0 | 0.1 |
| 58 | 10MS | 0 | 50.0 | 3.8 | 5.0 | 4.0 | 0.1 | 5MS | 0 | 62.5 | 4.8 | 2.5 | 2.0 | 0.1 |
| 59 | 60S | 10S | 450.0 | 36.3 | 35.0 | 35.0 | 0.2 | 60S | 60S | 800.0 | 75.2 | 60.0 | 60.0 | 0.2 |
| 60 | 10MS | 0 | 50.0 | 3.8 | 5.0 | 4.0 | 0.1 | 60S | 40S | 625.0 | 55.8 | 50.0 | 50.0 | 0.2 |
| 61 | 40S | 10S | 275.0 | 22.9 | 25.0 | 25.0 | 0.1 | 40S | 20S | 337.5 | 29.8 | 30.0 | 30.0 | 0.2 |

***P (Penultimate leaf)-one leaf below the flag leaf, F-flag leaf (In all the parameters mean value of both the leaves is given)**

****AUDPC-area under disease progress curve, rAUDPC-relative area under disease progress curve, FRS-final rust severity, CI-cofficient of infection**
