# Supplementary figures and images for: Residual effect of defeated stripe rust resistance genes/QTLs in bread wheat against prevalent pathotypes of Puccinia striiformis f. sp. tritici
Source: PLoS One. 2022 Apr 1;17(4):e0266482. doi: 10.1371/journal.pone.0266482 (PMC8975100; doi:10.1371/journal.pone.0266482)

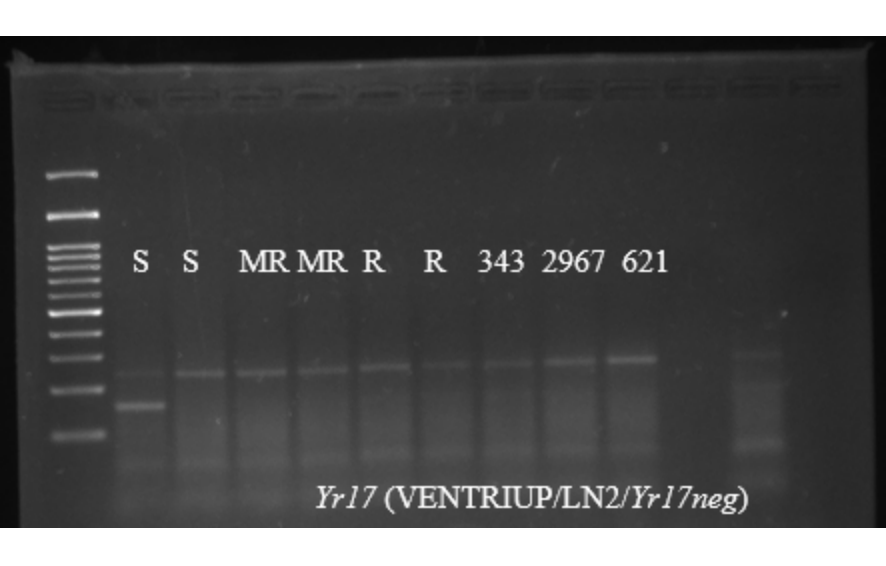

S S MR MR R R 343 2967 621

*Yr17* (VENTRIUP/LN2/*Yr17neg*)

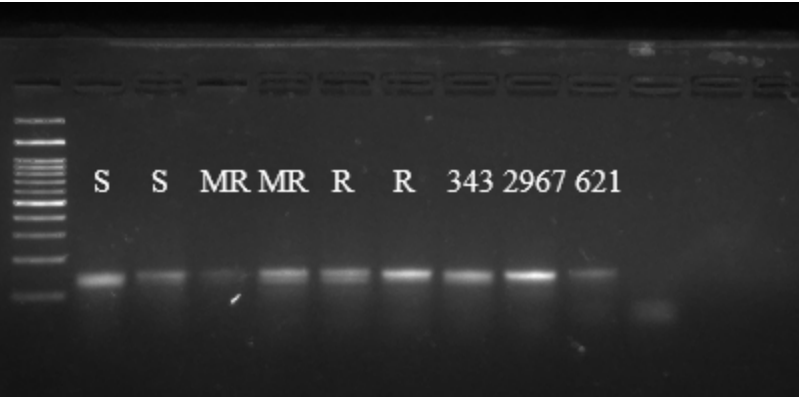

S S MR MR R R 343 2967 621

*Yr9* (Xgwm582-1B)

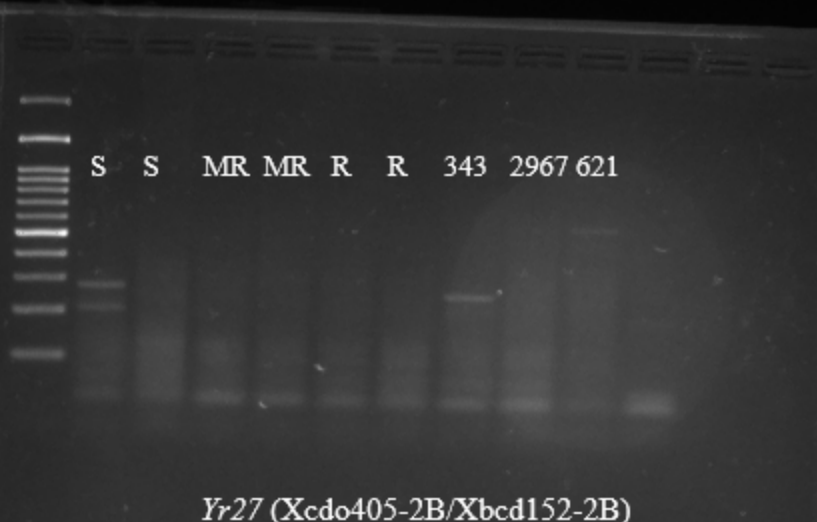

S S MR MR R R 343 2967 621

*Yr27* (Xcdo405-2B/Xbcd152-2B)

Supplement: S1 Raw images — (PDF) [file pone.0266482.s005.pdf]
