# Supplementary material for: Residual effect of defeated stripe rust resistance genes/QTLs in bread wheat against prevalent pathotypes of Puccinia striiformis f. sp. tritici
Source: PLoS One. 2022 Apr 1;17(4):e0266482. doi: 10.1371/journal.pone.0266482 (PMC8975100; doi:10.1371/journal.pone.0266482)
Supplement: Attachment — Submitted filename: Response to reviewers.docx [file pone.0266482.s006.docx]

**Revisions for Manuscript PONE-D-21-31839**

**Pointwise responses to the reviewer’s comments**

**Reviewer #1**

i) Key findings and Conclusion added in the Abstract.
ii) Needful has been done for the grammatical errors present in the manuscript.
iii) All the references have been arranged according to the PLOS format.

**Reviewer #2**

i) All the references have been arranged in the ascending order
ii) References has been cross checked in the main text. The missing references has been added in the Reference Section.
iii) Typographical mistakes have been corrected.

**Reviewer #3**

i) The abbreviations at the start of the sentence have been avoided.
ii) Discussion part has been revised.

**Additional requirements**

1. Manuscript has been written according to PLOS ONE’s standard style.
2. All the data underlying the findings is fully available in the Manuscript.
3. All the figures have been processed using PACE digital diagnostic tool to ensure that figures meet PLOS requirements.
4. All the references have been updated and corrected according to PLOS requirements.
