# Supplementary material for: Residual effect of defeated stripe rust resistance genes/QTLs in bread wheat against prevalent pathotypes of Puccinia striiformis f. sp. tritici
Source: PLoS One. 2022 Apr 1;17(4):e0266482. doi: 10.1371/journal.pone.0266482 (PMC8975100; doi:10.1371/journal.pone.0266482)
Supplement: Attachment — Submitted filename: Response to reviewers.docx [file pone.0266482.s007.docx]

**Revisions for Manuscript [PONE-D-21-31839R1]-[EMID:c3978ce058c1cd49]**

**Pointwise responses to the reviewer’s comments**

**Additional requirements**

1. All the figures have been processed using PACE digital diagnostic tool (in previous revision) to ensure that figures meet PLOS requirements.
2. All the references have been updated and corrected according to PLOS requirements.

**Changes made in references**:

- Reference no. 26 was clubbed with Reference no. 25, so it has been moved ahead.
- Reference no. 28 was clubbed with Reference no. 27, it has also been moved ahead.
- Other minor corrections in the references have been corrected.
- References has also been updated in Supplementary table 2 (S2_Table).
