# Supplementary material for: Residual effect of defeated stripe rust resistance genes/QTLs in bread wheat against prevalent pathotypes of Puccinia striiformis f. sp. tritici
Source: PLoS One. 2022 Apr 1;17(4):e0266482. doi: 10.1371/journal.pone.0266482 (PMC8975100; doi:10.1371/journal.pone.0266482)
Supplement: Attachment — Submitted filename: Response to reviewers.docx [file pone.0266482.s008.docx]

**Revisions for Manuscript [PONE-D-21-31839R2]-[EMID:** **f7ae2a847183cc53]**

**Pointwise responses to the reviewer’s comments**

**After careful reading of the paper following changes have been made and accordingly references have been updated.**

**Additional Editor Comments:**

The minor changes and information have been updated.

- Line23: **Pst** italicized
- Line47-49 updated: **83** permanently designated stripe rust resistance genes, **71** temporarily designated genes and **363** quantitative trait loci (QTLs); accordingly references have been updated.

**Additional requirements**

1. All the references have been updated and corrected according to PLOS requirements.

**Changes made in references**:

- Reference no. **5** and **6** have been removed and replaced with the latest updated references.

Deleted reference no. 5 and 6:

5.	McIntosh RA, Dubcovsky J, Rogers WJ, Morris C, Xia XC. Catalogue of gene symbols for wheat: 2017 supplement. [Internet]. 2017. Available from: <https://shigen.nig.ac.jp/wheat/komugi/genes/macgene/supplement2017.pdf>.

6.	[Gessese](about:blank) M, [Bariana](about:blank) H, Wong D, Hayden M, Bansal U. Molecular mapping of stripe rust resistance gene *Yr81* in a common wheat landrace Aus27430. Plant Dis. 2019; 103:1166-1171. doi: 10.1094/pdis-06-18-1055-re

Updated current references: (numbered as 5 and 6)

5.	McIntosh RA, Dubcovsky J, Rogers WJ, Xia XC, Raupp WJ. Catalogue of gene symbols for wheat: 2020 supplement. Annu Wheat Newslett. 2020; 66:109-149.

6.	Singh K, Batra R, Sharma S, Saripalli G, Gautam T, Singh R, et al. WheatQTLdb: a QTL database for wheat. Mol Genet Genom. 2021; 296(5):1051-1056. doi: 10.1007/s00438-021-01796-9

- Other minor corrections in the references have been corrected.
