# Supplementary material for: Residual effect of defeated stripe rust resistance genes/QTLs in bread wheat against prevalent pathotypes of Puccinia striiformis f. sp. tritici
Source: PLoS One. 2022 Apr 1;17(4):e0266482. doi: 10.1371/journal.pone.0266482 (PMC8975100; doi:10.1371/journal.pone.0266482)
Supplement: Attachment — Submitted filename: Response to reviewers.docx [file pone.0266482.s009.docx]

**Revisions for Manuscript [PONE-D-21-31839R3]-[EMID: df6535602fe5cb8a]**

**Pointwise responses to the reviewer’s comments**

**Journal Requirements:**

After reviewing all the references, one reference has been deleted and replaced with latest updated one.

**Deleted Reference no. 1**

1.	Bal RS. Effect of some fungicides and time of fungicidal spray on stripe rust of wheat. J Plant Pest Sci. 2014; 1:39-43.

**Updated latest reference no. 1**

2.	Singh H, Kaur J, Bala R, Singh S, Pannu PPS. Assessment of yield losses due to stripe rust caused by *Puccinia* *striiformis* f. sp. *tritici* on bread wheat cultivars in Punjab. Agric Res J. 2019; 56:698-702. doi: 10.5958/2395-146X.2019.00108.X

**All the remaining references are complete and correct.**

**Additional Editor Comments:**

No query from editor was received in this revision. All the queries of editor were addressed in previous revisions.

**Reviewer’s comments:**

All the queries from reviewer were addressed in the previous revisions.
